# Nail involvement in patients with epidermolysis bullosa: A systematic review

**DOI:** 10.1002/ski2.183

**Published:** 2022-11-10

**Authors:** Elena Pastrana‐Arellano, Diana Morales‐Olvera, María T. García‐Romero

**Affiliations:** ^1^ Dermatology Department National Institute of Pediatrics Mexico City Mexico

## Abstract

**Background:**

Nail changes in patients with congenital epidermolysis bullosa (EB) are caused by abnormalities of the nail matrix and bed secondary to pathogenic alterations of the dermoepidermal junction. Even though ungual alterations are extremely frequent in these patients, there are scarce studies about their frequency and/or association with subtypes or clinical course of EB.

**Objectives:**

To systematically review nail abnormalities in patients with EB reported in the literature.

**Methods:**

We searched all published articles in electronic databases until June 2020 reporting patients with EB with detailed descriptions of malformed/diseased nails using specific terms and inclusion/exclusion criteria. Clinical data were extracted by two independent authors. Descriptive statistics were used.

**Results:**

We included 36 articles reporting 74 individual patients with a mean age of 28.23 years: 29 (39.2%) had dominant dystrophic EB, 27 (36.4%) had junctional EB, 8 (10.8%) had EB simplex, 6 (8.1%) had Kindler syndrome and 4 (5.4%) had recessive dystrophic EB. The most common abnormalities were dystrophic nails (48.6%), anonychia (43.2%) and pachyonychia (40.5%). Anonychia was considered the most severe abnormality and was reported more frequently in patients with junctional (62.9%) and recessive dystrophic EB (50%). Multiple organ involvement was present in 52.7% of patients. Patients with severe junctional epidermolysis bullosa and recessive dominant epidermolysis bullosa presented anonychia since birth.

**Conclusions:**

In this summary of nail abnormalities in patients with EB, anonychia was more frequent in patients with severe EB subtypes and multiple organ involvement. Further prospective studies are required to understand the associations between nail abnormalities in specific EB subtypes and/or patient outcomes.

## Graphical abstract

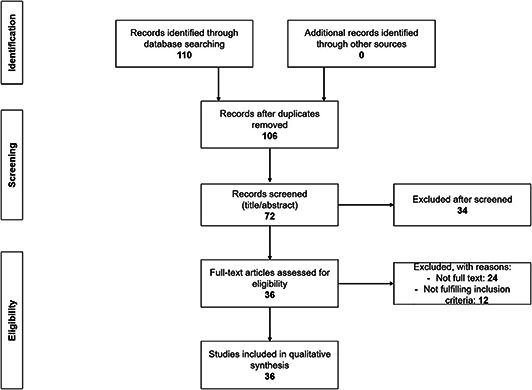

Nail changes in patient with congenital epidermolysis bullosa (EB) are a result of abnormalities of the nail matrix and bed secondary to pathogenic alterations of the dermoepidermal junction. They are extremely frequent, but there are scarce studies about their frequency and/or association with subtypes or clinical course. This systematic review of the literature found that all patients with EB have nail abnormalities, regardless of subtype. The most common ungual alterations were dystrophic nails (48.6%), anonychia (43.2%) and pachyonychia (40.5%). Anonychia was more frequent in patients with multiple organ involvement, and loss of multiple nails since birth or early life was reported in patients with severe subtypes.

1

**What is already known about this topic?**

- Nail changes in congenital epidermolysis bullosa (EB) are a result of abnormalities of the nail matrix and bed secondary to pathogenic alterations of the dermoepidermal junction.
- Ungual abnormalities are extremely frequent in patients with EB, but there are scarce studies about their frequency and/or association with subtypes or clinical course.

**What does this study add?**

- This systematic review of the literature found that all patients with EB have nail abnormalities, regardless of subtype. The most common ungual alterations were dystrophic nails (48.6%), anonychia (43.2%) and pachyonychia (40.5%).
- Anonychia was more frequent in patients with multiple organ involvement, and loss of multiple nails since birth or early life was reported in patients with severe subtypes.
- Nail examination in patients with EB can provide diagnostic and prognostic information.

## 2 INTRODUCTION

Congenital epidermolysis bullosa (EB) is a group of rare genetic diseases characterised by fragile skin and mucous membranes caused by mutations in genes that encode proteins involved in the structure and function of the dermoepidermal junction.^1^, ^2^

It is divided into four major types: simplex (EBS), junctional (JEB), dystrophic (DEB) and Kindler syndrome (KS). Subtypes are further sub‐classified by mode of inheritance, phenotype, immunofluorescence antigen mapping and genetic analysis findings.^3^, ^4^ Most EB subtypes, especially the more severe, involve organs and systems besides the skin and its appendages.

Clinical nail changes in EB are a result of abnormalities of the nail matrix and bed secondary to pathogenic alterations of the dermoepidermal junction and appear to be extremely common.^5^, ^6^ Surprisingly, little is known about the relationship between these and the type, subtype, severity and progression of the primary disease.

## 3 MATERIALS AND METHODS

**FIGURE 1:**
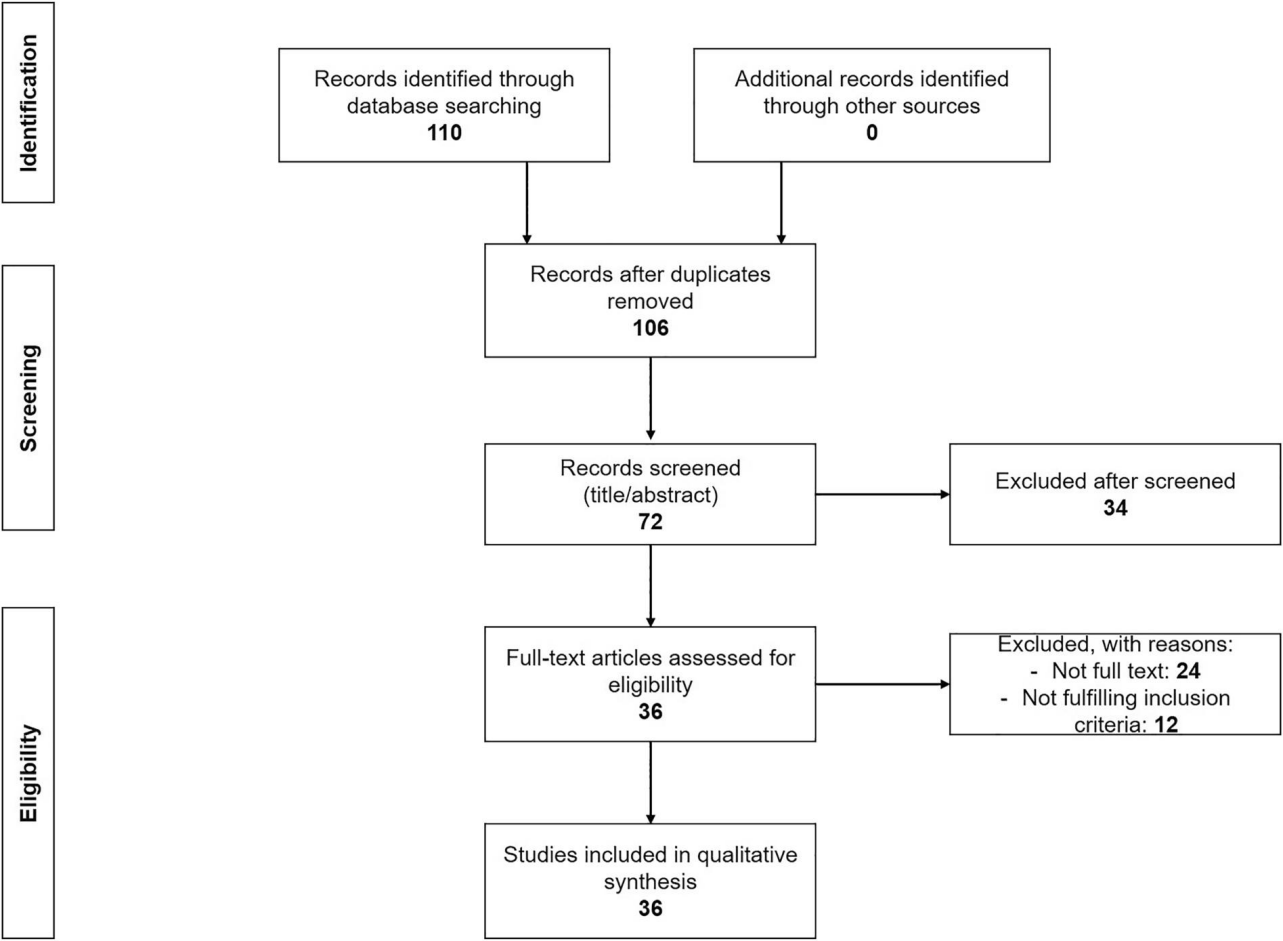
Flowchart of study selection process

We conducted a systematic review of the literature according to the Preferred Reporting Items for Systematic Reviews and Meta‐Analyses (PRISMA) guidelines in order to study nail involvement in patients with EB (Figure 1).

### 3.1 Literature search strategies

We searched for all relevant studies up until June 2020 in the following electronic databases: Medical Literature Analysis and Retrieval System Online (MEDLINE), Excerpta Medica dataBASE (EMBASE), Scientific Electronic Library Online (SciELO), World Health Organization (WHO), Literatura Latinoamericana y del Caribe en Ciencias de la Salud (LILACS), KoreaMed and Cochrane Central Register of Controlled Trials (CENTRAL) databases, without language or type of publication limitations.

The following search criteria were used: [(Epidermolysis Bullosa) NOT (Epidermolysis Bullosa Acquisita)] AND [(Nail Diseases) OR (Nails, Malformed)].

#### 3.1.1 Data extraction

Two authors (Diana Morales‐Olvera and Elena Pastrana‐Arellano) independently performed data extraction by examining titles, abstracts and full texts of the studies identified for eligibility. Any discrepancies were discussed; if uncertainty persisted, a third reviewer (María T. García‐Romero) evaluated the article.

#### 3.1.2 Inclusion criteria

We included all studies that detailed nail involvement in EB patients and that met the following criteria: (1) reported patients diagnosed with EB; (2) by either electron microscopy, immunofluorescence and/or genetic testing; and (3) with malformed or diseased nails who had at least one abnormality described.

#### 3.1.3 Exclusion criteria

Studies were excluded if there were no clear details regarding nail involvement in EB patients, if the studies or abstracts were unavailable, or were duplicated.

### 3.2 Outcome measures

The outcome of interest was the identification of at least one nail abnormality hypothesised to be generated by the pathogenesis of EB, as well as descriptions of the type, severity and number of nails that were involved.

The following data were extracted from the studies and collected in an electronic database: age, gender, ethnicity, EB subtype, family history, diagnostic method, genetic alteration (if known), number and location of affected nails, type of nail involvement (anonychia, pachyonychia, nail pigmentation, granulomatous tissue, discoloured nail, pincer nail deformity, parrot beak nail, rudimentary nail, ridged atrophic plates, subungual hyperkeratosis, long thick cuticles, onycholysis, dystrophic nails and/or other nail deformities), age of onset, and involvement of other organs or systems other than the skin (Table S1). EB subtypes were reclassified according to the most current classification^4^ based on the information described in the articles. Descriptive statistics were obtained using Microsoft Excel for Office 365Ⓡ, 2008.

## 4 RESULTS

Of the 110 articles retrieved from the electronic databases, 36 articles met the inclusion criteria (Figure 1)^7^, ^8^, ^9^, ^10^, ^11^, ^12^, ^13^, ^14^, ^15^, ^16^, ^17^, ^18^, ^19^, ^20^, ^21^, ^22^, ^23^, ^24^, ^25^, ^26^, ^27^, ^28^, ^29^, ^30^, ^31^, ^32^, ^33^, ^34^, ^35^, ^36^, ^37^, ^38^, ^39^, ^40^, ^41^. Most were case reports and small case series ≤6 patients, except for two^27^, ^41^ that reported 18 and 8 patients, respectively. In total, 74 relevant patient cases were included for data collection (Table S1).

Twenty‐nine patients (39.2%) had dominant DEB (21 pruriginosa and 8 localised), 27 (36.4%) had JEB (16 intermediate, 3 laringo‐onycho‐cutaneous syndrome (LOC), 2 localised, 3 severe, 2 with nephrotic syndrome and interstitial lung disease, and 1 with pyloric atresia), 8 (10.8%) had EBS (5 severe, 1 had localised with nephropathy, 1 had mottled pigmentation and 1 had intermediate with muscular dystrophy), 6 (8.1%) had KS and 4 (5.4%) recessive DEB (2 had localised, 1 severe and 1 unclassifiable). The mean age of patients was 28.23 years (SD ± 21.52), and 43 patients (58.1%) were male. Genetic testing was the most common diagnostic method (59.4%). The most common abnormalities were dystrophic nails (48.6%), anonychia (43.2%) and pachyonychia (40.5%). Combined fingernail and toenail involvement occurred in 45.9% of the patients. On average, 13.8 (SD ± 6.9) nails were affected. Other organ or system involvement was present in 39 patients (52.7%); the oral cavity was the most frequently affected site (71.7%) followed by ophthalmologic and gastrointestinal involvement (each 38.4%) (Table 1).

**TABLE 1:** Clinical characteristics of patients included in the review categorised by epidermolysis bullosa (EB) subtype

	All subtypes; *n* = 74 (100%)	EBS; *n* = 8 (10.8%)	JEB; *n* = 27 (36.4%)	DDEB; *n* = 29 (39.2%)	RDEB; *n* = 4 (5.4%)	KS; *n* = 6 (8.1%)
Age in years, mean (SD)	28.2 (21.5)	16 (15.4)	21.1 (16.7)	34.87 (21.96)	34 (38.6)	36.3 (21.3)
Gender distribution						
Female sex, *n* (%)	31 (41.8%)	2 (25%)	11 (40.7%)	14 (48.3%)	2 (50%)	2 (33.3%)
Ethnicity						
Caucasian, *n* (%)	4 (5.4%)	1 (12.5%)	2 (7.4%)	1 (3.4%)		
Taiwanese, *n* (%)	1 (1.3%)	1 (12.5%)				
Saudi Arabian, *n* (%)	1 (1.3%)	1 (12.5%)				
Pakistani, *n* (%)	3 (4%)		3 (11.1%)			
Omani, *n* (%)	2 (2.7%)		2 (7.4%)			
Australian, *n* (%)	8 (10.8%)		8 (29.6%)			
Chinese, *n* (%)	21 (28.3%)			21 (72.4%)		
Vietnamese, *n* (%)	1 (1.3%)			1 (3.4%)		
Italian, *n* (%)	2 (2.7%)				2 (50%)	
Japanese, *n* (%)	2 (2.7%)			2 (6.8%)		
Turkish, *n* (%)	4 (5.4%)		3 (11.1%)		1 (25%)	
Maltese, *n* (%)	1 (1.3%)			1 (3.4%)		
Moroccan, *n* (%)	1 (1.3%)		1 (3.7%)			
Yemeni, *n* (%)	1 (1.3%)		1 (3.7%)			
Palestinian, *n* (%)	5 (6.7%)					5 (83.3%)
Not reported, *n* (%)	17 (22.9%)	5 (62.5%)	7 (25.9%)	3 (10.3%)	1 (25%)	1 (16.7%)
Diagnostic method						
Electron microscopy, *n* (%)	37 (50%)	8 (100%)	22 (81.4%)	5 (17.2%)	2 (50%)	0
Immunofluorescence, *n* (%)	34 (45.9%)	3 (37.5%)	21 (77.7%)	6 (20.7%)	3 (75%)	1 (16.6%)
Genetic testing, *n* (%)	44 (59.4%)	3 (37.5%)	9 (33.3%)	25 (86.2%)	2 (50%)	5 (83.3%)
H‐E/immunohistochemistry, *n* (%)	16 (21.6%)	1 (12.5%)	9 (33.3%)	3 (10.3%)	2 (50%)	1 (16.6%)
Other organ/system involvement, *n* (%)	39 (52.7%)	6 (75%)	24 (88.8%)	1 (3.4%)	2 (50%)	6 (100%)
Oral, *n* (%)	28 (71.7%)	4 (66.6%)	18 (66.6%)	0	1 (50%)	5 (83.3%)
Ophthalmologic, *n* (%)	15 (38.4%)	2 (33.3%)	8 (29.6%)	0	0	5 (83.3%)
Gastrointestinal, *n* (%)	15 (38.4%)	2 (33.3%)	6 (22.2%)	1 (100%)	1 (50%)	5 (83.3%)
Nutritional, *n* (%)	8 (20.5%)	2 (33.3%)	5 (18.5%)	0	0	1 (16.6%)
Genitourinary, *n* (%)	8 (20.5%)	1 (16.6%)	3 (11.1%)	0	0	4 (66.6%)
Musculoskeletal, *n* (%)	7 (17.9%)	1 (16.6%)	0	0	0	6 (100%)
Nutritional deficiency, *n* (%)	8 (20.5%)	2 (33.3%)	5 (18.5%)	0	0	1 (16.6%)
Number of nails involved, mean (SD)	13.8 (6.9)	10.8 (9)	17 (5.9)	11.1 (5.7)	10.2 (6.8)	NR
Type of nail involvement						
Dystrophic nail, *n* (%)	36 (48.6%)	4 (50%)	15 (55.5%)	10 (34.5%)	2 (50%)	5 (83.3%)
Anonychia, *n* (%)	32 (43.2%)	3 (37.5%)	17 (62.9%)	10 (34.5%)	2 (50%)	0
Pachyonychia, *n* (%)	30 (40.5%)	2 (25%)	15 (55.5%)	13 (44.8%)	0	0
Hypoplastic nails, *n* (%)	15 (20%)	2 (25%)	2 (7.4%)	9 (31%)	2 (50%)	0
Discoloured nails, *n* (%)	9 (12.1%)	0	9 (33.3%)	0	0	0
Granulomatous tissue, *n* (%)	4 (5.4%)	0	4 (14.8%)	0	0	0
Pigmentation, *n* (%)	3 (4%)	2 (25%)	0	1 (3.4%)	0	0
Pincer nail deformity, *n* (%)	3 (4%)	1 (12.5%)	1 (3.7%)	1 (3.4%)	0	0
Rudimental nails, *n* (%)	3 (4%)	0	1 (3.7%)	1 (3.4%)	1 (25%)	0
Parrot beak nail, *n* (%)	1 (1.3%)	0	1 (3.7%)	0	0	0
Ridged atrophic plate, *n* (%)	1 (1.3%)	0	0	1 (3.4%)	0	0
Distal loss of the nail, *n* (%)	1 (1.3%)	0	0	1 (3.4%)	0	0
Subungual hyperkeratosis, *n* (%)	1 (1.3%)	0	1 (3.7%)	0	0	0
Long thick cuticle, *n* (%)	1 (1.3%)	0	0	0	0	1 (16.6%)
Onychomadesis, *n* (%)	1 (1.3%)	1 (12.5%)	0	0	0	0
Onycholysis, *n* (%)	1 (1.3%)	0	1 (3.7%)	0	0	0
Other nail deformity, *n* (%)	3 (4%)	1 (12.5%)	0	2 (6.9%)	0	0

Abbreviations: DDEB, dystrophic dominant epidermolysis bullosa; EBS, epidermolysis bullosa simplex; JEB, junctional epidermolysis bullosa; KS, Kindler syndrome; RDEB, recessive dominant epidermolysis bullosa.

**FIGURE 2:**
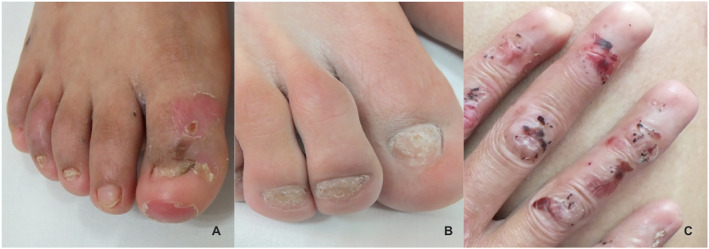
Clinical images representing nail alterations in epidermolysis bullosa (EB). (a) Dystrophic nails in a patient with recessive dominant epidermolysis bullosa (RDEB). (b) Hypoplastic nails in a patients with dystrophic dominant epidermolysis bullosa (DDEB). (c) Anonychia of all nails in a patient with RDEB

Anonychia was the most severe nail abnormality and was present in 62.9% of the patients with JEB, 50% of the patients with recessive (RDEB) (Figure 2c), 37.5% with EBS, and 34.5% of the patients with dominant (DDEB). All patients with severe JEB, RDEB and DDEB had anonychia since birth.

In patients with EBS, the most frequently reported nail alteration was dystrophic nails (50%). Four (80%) patients with EBS severe and nail alterations presented involvement of other organs, the oral cavity was the most frequently affected site.

Among patients with JEB, anonychia was present in 62.9% and pachyonychia and dystrophic nails was present in 55.5% of the patients. The 3 patients with severe JEB presented anonychia since the neonatal period, 2 also had periungual granulomatous tissue. Patients with syndromic JEB/interstitial lung disease and nephrotic syndrome had pachyonychia more frequently (83.3%). Of all of the JEB patients, 88.8% presented other organ involvement, and the oral cavity was the most frequently affected. Among the 17 patients with anonychia, 15 patients had multiorgan involvement (88.2%), and 12 of the 15 patients with dystrophic nails had multiorgan involvement (80%).

In 27 patients with DDEB, the most frequent abnormalities included pachyonychia in 44.4%, and anonychia and dystrophic nails in 34.5% each. Only one patient with DDEB had gastrointestinal involvement, in 9 no other organs were involved, and in 17 no information was provided. All patients with RDEB had nail alterations: anonychia, dystrophic and hypoplastic nails were each reported in two of the 4 patients (Figure 2a,b). One patient with severe RDEB had anonychia of 20 nails since birth. The 2 patients with RDEB and anonychia had gastrointestinal and oral involvement each.

Among patients with KS, 83.3% had dystrophic nails, and 100% of the patients had other organs affected. All had musculoskeletal involvement, and 83.3% also had ophthalmologic, oral and gastrointestinal manifestations.

## 5 DISCUSSION

Nail involvement is a frequent clinical finding in EB patients since antigenic expressions of the basement membrane zone components in the matrix, nail bed, proximal nail fold, and hyponychium are similar to normal skin.^43^ It can be the first (or only) sign of the disease, as in the nail‐only DDEB and EBS subtypes.^42^, ^43^ In this systematic review, the most frequently reported nail abnormalities in EB patients were dystrophic nails (48.6%) and anonychia (43.2%).

In patients with EBS we confirmed already reported alterations^43^, ^44^ as well as novel ones like brown pigmentation and hypoplastic nails.^7^, ^12^, ^38^ We found patients with EBS severe had anonychia and dystrophic nails more frequently, and all nail changes appeared before 2 years of age. Even though the absence of nail abnormalities has commonly been used in the past to exclude a diagnosis of this subtype,^44^ in this study half of patients with EBS had nail involvement and those with EBS severe subtypes had nail alterations before 2 years of age.

The previously reported nail involvement in patients with JEB includes onycholysis, pachyonychia, abnormal shape, granulation tissue and anonychia.^5^, ^43^ Severe nail dystrophy has been reported in the literature, affecting up to 90% of patients.^44^ We confirmed such nail alterations, but nail discolouration was a novel abnormality that was found in 33.3% of the patients with JEB^40^, ^41^ and only 55.5% exhibited dystrophy. Other characteristics that were observed in our review but that had never been previously described included hypoplastic nails and subungual hyperkeratosis^23^, ^41^ Even though time of onset was not reported in all patients, 7 patients with JEB had nail abnormalities since birth; and 2 patients with JEB severe, one with JEB intermediate and one with JEB localised had anonychia of 20 nails since birth or neonatal period. Hence, this finding could provide useful bedside diagnostic and prognostic information.

Laringo‐onychocutaneous (LOC) syndrome is a special JEB subtype in which nail abnormalities have been reported in 100% patients: dystrophic nails, pachyonychia, nail erosions with granulation tissue and/or anonychia.^43^ In our literature review, 3 patients with this subtype were identified, and their nail findings were similar, except for dystrophic nails. Parrot beak nail was present in 1 patient^22^ and had only been previously described in KS syndrome, not in LOC syndrome.

Nail abnormalities in DEB can range from minor nail dystrophy to complete anonychia in the most severe subtypes.^5^ Permanent nail loss is due to the destruction of the nail matrix secondary to repeated extensive sub‐basement membrane blistering and scarring.^5^, ^44^ The most frequent DEB subtype that was observed in this systematic review was DDEB pruriginosa, patients had anonychia (42.8%), pachyonychia (38%), hypoplastic nails (23.8%), dystrophic nails (14.2%) and pincer nail deformities (4.7%). Alterations that have been previously described include nail thickening and anonychia as well as dystrophic nails.^27^, ^43^ Both DDEB and RDEB have been associated with nail thickening in >75% patients.^43^ Our patients with DDEB localised had dystrophic nails, pachyonychia and hypoplastic nails in 87.5%, 62.5% and 50%.

The only included patient with RDEB severe had anonychia of all nails since birth.^34^ One patient with RDEB that we couldn't further classify had hypoplastic nails and anonychia of 5 nails. The nail characteristics that we observed in 2 related patients with RDEB localised (one had very pruriginous lesions and the other had ¨nails only¨^18^ disease) were rudimental or hypoplastic and dystrophic nails, not consistent with previously reported findings of nail thickening and anonychia.^43^

Nail changes in KS are uncommon and diverse.^1^, ^43^, ^45^ In this review, 5/6 patients with KS had dystrophic nails and one had long and thick cuticles.^17^ The pathogenesis of long cuticles is not yet understood. Different theories that explain this pathogenesis include the atrophy of the “living” layers of the skin in KS and a relative sparing of the “dead” horny layer, which forms the cuticle of the nail.^45^

Nail alterations in EB may be related to the severity of each subtype phenotype or specific genotypes.^43^ In this review, anonychia of multiple nails at birth or neonatally was reported in patients with severe JEB and RDEB and could be a useful clinical finding to suspect these severe subtypes. They may also be helpful in predicting systemic involvement: approximately half of the patients included in this review presented multiple organ involvement, and anonychia was more common in these patients than in those without it.

This review had some limitations, due to its retrospective nature. Data were collected from observational studies subject to inherent biases, including wide variability in information reported and unexplained findings. Many of the articles included in this review did not specify EB subtypes, and/or provide genetic information. We included a moderate number of published cases that may not represent the real prevalence or actual clinical settings of nail alterations found in specific EB subtypes. Finally, both ageing and nutrient deficiencies may also play a role in nail health and integrity in patients with EB.

Nevertheless, this study is the first to systematically review and provide a high‐level summary of the clinical characteristics of nail abnormalities in EB patients. The rarity of EB, as well as the fact that nail involvement is a complication that has not been previously systematically reviewed, makes it a challenging topic to investigate.

Nail changes in EB patients are frequent and diverse and may be useful in diagnosing the disease as well as providing prognostic information about severity of and multiorganic involvement. Nails should be examined and nail involvement documented at baseline and during routine follow‐up appointments. Additional prospective studies are needed to provide more information about time of onset of nail abnormalities, correlation with the genotype‐phenotype of EB subtypes and their association with patient outcomes.

## CONFLICTS OF INTEREST

The author declares that there is no conflict of interest that could be perceived as prejudicing the impartiality of the research reported.

## AUTHOR CONTRIBUTIONS

**Elena Pastrana‐Arellano:** Data curation (equal); Formal analysis (equal); Investigation (equal); Writing – original draft (equal). **Diana Morales‐Olvera:** Data curation (supporting); Formal analysis (supporting); Investigation (supporting); Writing – original draft (equal). **Maria T. Garcia‐Romero:** Conceptualization (lead); Formal analysis (lead); Investigation (lead); Methodology (lead); Resources (lead); Validation (lead); Writing – review & editing (lead).

## ETHICS STATEMENT

This research protocol was approved by our institutional review board and registered with the number C022‐2019.

## Supporting information

Supporting Information S1

## Data Availability

The data that support the findings of this study are available from the corresponding author upon reasonable request.
