# Supplementary material for: Nail involvement in patients with epidermolysis bullosa: A systematic review
Source: Skin Health Dis. 2022 Nov 10;3(1):e183. doi: 10.1002/ski2.183 (PMC9892443; doi:10.1002/ski2.183)
Supplement: Supplementary file 1 — Supporting Information S1 [file SKI2-3-e183-s001.docx]

**Supplemental Table 1**. All included articles and all patients´ demographic and clinical information.

| **First author and year** | **Case no.** | **Age** | | **Sex** | **Ethnicity** | **EB subtype** | **Family history** | **Diagnostic methods: 1=EM**  **2=IF 3=Genetic testing**  **4= IHC** | **Genetic mutation** | **No. of nails affected** | **Finger/ toenails or both** | **Age at onset** | **Type of nail involvement** | **Other involvement** |
| --- | --- | --- | --- | --- | --- | --- | --- | --- | --- | --- | --- | --- | --- | --- |
| **Vahidnezhad H, 2018** | 1 | 33 | M | | Caucasian | EBS localized with nephropathy AR | N | 1,2,3 | CD151 | 20 | Both | NR | Pachyonychia, longitudinal pigmentation and hypoplastic nails | Ophthalmologic, oral, gastrointestinal, nutritional deficiency, renal and alopecia. |
| **Colombo EA, 2010** | 2 | 13 | F | | Caucasian | JEB with interstitial lung disease and nephrotic syndrome  AR | Y | 3 | ITGA3 | NR | NR | NR | Pachyonychia | Ophthalmologic, oral, nutritional deficiency, pulmonary, and cardiac |
|  | 3 | 9 | M | | Caucasian | JEB with interstitial lung disease and nephrotic syndrome AR | Y | 3 | ITGA3 | 10 | Foot | NR | Pachyonychia | Nutritional deficiency, pulmonary and cardiac |
| **Turcan I, 2016** | 4 |  | M | | NR | JEB localized  ( AD) | Y | 1 ,2, 3 | ITGB4 | 20 | Both | Birth | Dystrophic nails and anonychia | Ophthalmologic, genitourinary and otorhinolaryngological |
| **Chen Q, 2015** | 5 | 68 | M | | Chinese | DDEB pruriginosa | Y | 3 ,4 | COL7A1 | NR | Both | 30´s | Dystrophic nails | None |
| **El Hachem, 2015** | 6 | 49 | M | | Palestinian | KS AR | Y | 2, 3 | FERMT1 | NR | NR | NR | Dystrophic nails | Ophthalmologic, oral, gastrointestinal, musculoskeletal and genitourinary |
|  | 7 | 39 | M | | Palestinian | KS | Y | 3 | FERMT1 | NR | NR | NR | Dystrophic nails | Ophthalmologic, oral, gastrointestinal and musculoskeletal |
|  | 8 | 19 | M | | Palestinian | KS | Y | 3 | FERMT1 | NR | NR | NR | Dystrophic nails | Ophthalmologic, oral, gastrointestinal, musculoskeletal and genitourinary |
|  | 9 | 44 | M | | Palestinian | KS | Y | 3 | FERMT1 | NR | NR | NR | Dystrophic nails | Ophthalmologic, oral, gastrointestinal, musculoskeletal and genitourinary |
|  | 10 | 63 | F | | Palestinian | KS | Y | 3 | FERMT1 | NR | NR | NR | Dystrophic nails | Ophthalmologic, oral, gastrointestinal, musculoskeletal and genitourinary |
| **El Darouti MA, 2015** | 11 | 17 | M | | NR | EBS severe?/ Kallin syndrome (deafness, alopecia, anodoncia, nail dystrophy) | N | 1, 4 |  | 20 | Both | NR | Anonychia, pachyonychia and pigmentation | Oral, otorhinolaryngological and alopecia |
| **Rosmaninho A, 2014** | 12 | 0 | F | | NR | RDEB no information to further subclassify, complete absence of colVII | N | 2, 4 |  | 5 | Foot | At birth | Hypoplastic and anonychia | Gastrointestinal |
| **Diociaiuti A, 2013** | 13 | 8 | F | | NR | JEB with pyloric atresia AR | N | 1, 2, 3 | ITGB4 | NR | Hand | Neonatal period | Dystrophic nails | Gastrointestinal and genitourinary |
| **Almeida HL, 2012** | 14 | 46 | F | | NR | DDEB localized | Y | 1, 2, 3 | COL7A1 | 19 | Both | Since childhood | Dystrophic nails | None |
| **Brick K, 2012** | 15 | NR | F | | Caucasian | DDEB pruriginosa | Y | 3 | COL7A1 | 20 | Hand | NR | Pachyonychia and pincer nail deformity | Gastrointestinal |
| **Gupta V, 2011** | 16 | 4 | F | | NR | KS | N | 4 |  | NR | Both | NR | Long and thick cuticles | Nutritional deficiency and musculoskeletal |
| **Pruneddu S, 2011** | 17 | 69 | F | | Italian | RDEB localized pruriginosa | Y | 1 ,2, 3 | COL7A1 | 10 | Foot | NR | Dystrophic and rudimental nails | None |
|  | 18 | 66 | M | | Italian | ¨Nails-only¨ localized RDEB | Y | 1 ,2, 3 | COL7A1 | 6 | Foot | NR | Dystrophic and hypoplastic nails | None |
| **Pérez A, 2010** | 19 | 56 | M | | NR | JEB intermediate | N | 2 |  | 20 | Both | NR | Anonychia | Oral |
| **Andres C, 2009** | 20 | NR | M | | NR | EBS with mottled pigmentation AD | Y | 1, 3 | KRT5 | 3 | Hand | NR | Dystrophic nails | None |
| **Rizzo C, 2008** | 21 | 47 | F | | Vietnamese | DDEB localized | Y | 2, 3, 4 | NOT REPORTED | 20 | Both | childhood | Dystrophic and rudimentary with ridged atrophic plates and distal loss of the nails | None |
| **Kim CC, 2007** | 22 | 12 | F | | Pakistani | JEB (LOC syndrome) AR | Y | 1 ,2, 3 | LAMA3A | 15 | Both | NR | Anonychia, pachyonychia and parrot beak nail | Ophthalmologic, oral, gastrointestinal and hematology |
| **Pasmooij AM, 2007** | 23 | 7 | M | | NR | JEB intermediate | N | 1, 2, 3 | COL17A1 | NR | Hand | NR | Anonychia and dystrophic nails | None |
|  | 24 | 43 | F | | NR | JEB intermediate | Y | 1, 2, 3 | COL17A1 | NR | Both | NR | Anonychia and dystrophic nails | Oral and alopecia |
|  | 25 | 38 | M | | Turkish | JEB localized | Y | 1, 2, 3 | COL17A1 | NR | Both | NR | Dystrophic nails, subungual hyperkeratosis, pachyonychia and hypoplastic nails | Oral |
| **Casanova JM, 2006** | 26 | 0 | M | | Moroccan | JEB severe | N | 1, 2 |  | 3 | Hand | at birth | Anonychia and granulomatous nail bed | Nutritional deficiency and hematology |
| **Mitsui H, 2005** | 27 | 41 | M | | NR | JEB intermediate | NR | 1, 2, 3 | COL17A1 | 20 | Both | neonatal period | Dystrophic nails and anonychia | Oral and alopecia |
| **Sato-Matsumura KC, 2002** | 28 | 35 | M | | Japanese | DDEB localized nails only | Y | 3 | COL17A1 | NR | Foot | childhood | Pachyonychia, hypoplastic and dystrophic nails: brown pigmentation, nail plates buried in the nail bed | None |
|  | 29 | 70 | F | | Japanese | DDEB localized nails only | Y | 3 | COL7A1. | 10 | Foot | childhood | Pachyonychia, hypoplastic and dystrophic nails: nail plates buried in the nail bed | None |
| **Jiang W, 2002** | 30 | 72 | F | | Chinese | DDEB pruriginosa | Y | 3 | COL7A1 | NR | Foot | NR | Hypoplastic nails | NS |
|  | 31 | 66 | F | | Chinese | DDEB pruriginosa | Y | 3 | COL7A1 | NR | Foot | NR | Anonychia | NS |
|  | 32 | 48 | M | | Chinese | DDEB pruriginosa | Y | 3 | COL7A1 | NR | NR | NR | Anonychia | NS |
|  | 33 | 41 | F | | Chinese | DDEB pruriginosa | Y | 3 | COL7A1 | NR | Foot | NR | Anonychia | NS |
|  | 34 | 50 | M | | Chinese | DDEB pruriginosa | Y | 3 | COL7A1 | NR | NR | NR | Pachyonychia | NS |
|  | 35 | 63 | F | | Chinese | DDEB pruriginosa | Y | 3 | COL7A1 | NR | Both | NR | Pachyonychia and anonychia | NS |
|  | 36 | 62 | F | | Chinese | DDEB pruriginosa | Y | 3 | COL7A1 | NR | NR | NR | Anonychia | NS |
|  | 37 | 1 | F | | Chinese | DDEB pruriginosa | Y | 3 | COL7A1 | NR | NR | NR | Hypoplastic nails | NS |
|  | 38 | 13 | M | | Chinese | DDEB pruriginosa | Y | 3 | COL17A1 | NR | Foot | NR | Anonychia | NS |
|  | 39 | 13 | M | | Chinese | DDEB pruriginosa | Y | 3 | COL7A1 | NR | NR | NR | Hypoplastic | NS |
|  | 40 | 22 | F | | Chinese | DDEB pruriginosa | Y | 3 | COL7A1 | NR | Foot | NR | Anonychia | NS |
|  | 41 | 35 | M | | Chinese | DDEB pruriginosa | Y | 1, 2, 3 | COL7A1 | NR | Both | NR | Pachyonychia and anonychia | None |
|  | 42 | 42 | M | | Chinese | DDEB pruriginosa | Y | 3 | COL7A1 | NR | NR | NR | Pachyonychia | NS |
|  | 43 | 38 | M | | Chinese | DDEB pruriginosa | Y | 3 | COL7A1 | NR | NR | NR | Pachyonychia | NS |
|  | 44 | 29 | F | | Chinese | DDEB pruriginosa | Y | 3 | COL7A1 | NR | Both | NR | Pachyonychia and hypoplastic | NS |
|  | 45 | 17 | M | | Chinese | DDEB pruriginosa | Y | 3 | COL7A1 | NR | Foot | NR | Dystrophic nails | NS |
|  | 46 | 10 | F | | Chinese | DDEB pruriginosa | Y | 3 | COL7A1 | NR | Foot | NR | Hypoplastic nails | NS |
|  | 47 | 13 | M | | Chinese | DDEB pruriginosa | Y | 3 | COL7A1 | NR | NR | NR | Pachyonychia | NS |
| **Ning CC, 2001** | 48 | 0 | M | | Taiwanese | EBS severe | Y | 1, 3 | KRT14 | NR | Hand | 0 | Onychomadesis and other nail deformity (shedding of the nails) | None |
| **Parsapour K, 2001** | 49 | 0,04 | M | | Yemeni | Severe JEB | N | 2 |  | 20 | Both | At birth | Anonychia and granulomatous nail bed | Gastrointestinal, nutritional deficiency, hematology and pulmonary |
| **Dharma B, 2001** | 50 | 0,58 | M | | NR | DDEB localized nails only | Y | 3 | COL7A1 | 3 | Both | NR | Pachyonychia and hypoplastic | None |
| **Kim DK, 2000** | 51 | 4 | M | | NR | EBS severe | Y | 1, 2 |  | 1 | Hand | At birth | Dystrophic nails | Gastrointestinal and genitourinary |
| **Tang WY, 1999** | 52 | 17 | M | | Chinese | DEB localized (likely DDEB) | Y | 1, 2 |  | 10 | Foot | NR | Pachyonychia, anonychia, hypoplastic and dystrophic nails | None |
|  | 53 | 12 | M | | Chinese | DEB localized (likely DDEB) | Y | 1, 2 |  | NR | Foot | NR | Dystrophic nails | None |
| **Swensson O, 1988** | 54 | 39 | M | | NR | JEB intermediate AD | Y | 1 |  | 20 | Both | 4 | Anonychia | Oral, alopecia and squamous cell cancer |
|  | 55 | 32 | M | | NR | JEB intermediate AD | Y | 1 |  | NR | Both | NR | Anonychia and dystrophic nails | Oral, alopecia and squamous cell cancer |
| **Gutzmer R, 1997** | 56 | 1,25 | M | | Turkish | Severe RDEB | N | 4 |  | 20 | Both | At birth | Anonychia | Oral |
|  | 57 | 0 | F | | Turkish | Severe JEB | N | 4 |  | 20 | Both | At birth | Anonychia | Oral |
| **Cambiaghi S, 1997** | 58 | 19 | F | | NR | DDEB Pruriginosa | N | 1, 2 |  | 10 | Foot | NR | Anonychia and dystrophic nails | None |
| **Tay YK, 1996** | 59 | 6 | F | | NR | EBS severe | N | 1, 2 |  | NR | Both | 2 | Pincer nail deformity | Oral |
| **Phillips RJ, 1994** | 60 | 3 | F | | Pakistani | JEB (LOC syndrome) | N | 1 |  | 4 | Both | NR | Pachyonychia and granulomatous tissue on the nail beds | Ophthalmologic oral and hematology |
|  | 61 | 0,33 | M | | Pakistani | JEB (LOC syndrome) | NR | 1, 2 |  | NR | Both | Infant period | Pachyonychia and granulomatous tissue on the nail beds | Ophthalmologic, oral, nutritional deficiency, hematology and genitourinary |
| **Abanmi A, 1994** | 62 | NR | M | | Saudi Arabian | EBS severe | Y | 1 |  | 10 | Foot | 3 months | Anonychia, hypoplastic and dystrophic nails | Ophthalmologic, oral, nutritional deficiency and hematology |
| **Niemi KM, 1988** | 63 | 36 | F | | NR | EBS intermediate with muscular dystrophy likely AR | Y | 1 |  | NR | Both | NR | Anonychia and dystrophic nails | Musculoskeletal |
| **Heagerty AH, 1985** | 64 | 20 | F | | Omani | JEB intemediate | Y | 1, 2 |  | NR | Both | NR | Anonychia, pachyonychia, discolored nails, pincer nail deformity | Oral |
|  | 65 | 21 | F | | Omani | JEB intermediate | Y | 1, 2 |  | NR | Both | NR | Anonychia, pachyonychia, discolored nails and dystrophic nails | None |
| **Hintner H, 1982** | 66 | 40 | M | | Australian | JEB intermediate | Y | 1, 2 |  | 20 | Both | NR | Dystrophic, pachyonychia, anonychia and discolored nails | Oral, gastrointestinal and alopecia |
|  | 67 | 37 | M | | Australian | JEB intermediate | Y | 1,2 |  | 20 | Both | NR | Anonychia, pachyonychia, discolored nails and dystrophic nails | Ophthalmologic, oral, gastrointestinal and alopecia |
|  | 68 | 38 | F | | Australian | JEB intermediate | Y | 1, 2 |  | 20 | Both | NR | Anonychia, pachyonychia, discolored nails, and dystrophic nails | Ophthalmologic, oral, gastrointestinal and alopecia |
|  | 69 | 33 | F | | Australian | JEB intermediate | Y | 1, 2 |  | 20 | Both | NR | Anonychia, pachyonychia, discolored nails and dystrophic nails | Ophthalmologic, oral and alopecia |
|  | 70 | 17 | M | | Australian | JEB intermediate | N | 1, 2 |  | 20 | Both | NR | Pachyonychia, discolored and dystrophic nails | Oral, otorhinolaryngological and alopecia |
|  | 71 | 25 | M | | Australian | JEB intermediate | N | 1, 2 |  | 20 | Both | NR | Pachyonychia, discolored and dystrophic | Alopecia |
|  | 72 | 14 | F | | Australian | JEB intermediate | N | 1, 2 |  | NR | Both | NR | Onycholysis, rudimentary nails and hypoplastic | Oral |
|  | 73 | 3 | M | | Australian | JEB intermediate | N | 1, 2 |  | NR | Foot | NR | Discolored nails, pachyonychia and dystrophic nails | None |
| **Jones RR, 1979** | 74 | 27 | M | | Maltese | DDEB localized | Y | 4 |  | 10 | Foot | NR | Pachyonychia and dystrophic nails | None |

M= male, F= female, Y= yes, N= no, NS= Not specified, EM= electron microscopy, IF= immunofluorescence, IHC= immunohistochemistry, EBS= epidermolysis bullosa simplex, JEB= junctional epidermolysis bullosa, DDEB= dominant dystrophic epidermolysis bullosa, RDEB= recessive dominant epidermolysis bullosa, KS= Kindler síndrome, LOC= laringo-onycho-cutaneous síndrome
